# Ras Participates in the Regulation of the Stability of Adenoviral Protein E1A via MAP-kinase ERK

**DOI:** 10.32607/actanaturae.11675

**Published:** 2022

**Authors:** A. V. Morshneva, O. O. Gnedina, D. N. Kindt, M. V. Igotti

**Affiliations:** Institute of Cytology, Russian Academy of Sciences, St. Petersburg, 194064 Russia

**Keywords:** molecular oncology, Ras, E1A, combination therapy, histone deacetylase inhibitors

## Abstract

The E1A adenoviral protein required for the initiation of the viral life cycle is being actively studied as a sensitizing agent in the combination therapy of cancer, and tumors with activated Ras in particular. We investigated the role played by the Ras signaling pathway in the regulation of E1A protein stability and showed that overexpression of activated Ras increases the basal level of E1A, but enhances the degradation of the E1A protein under treatment with histone deacetylase inhibitors (HDIs). It has been found that the MAP kinase ERK is the key factor in E1A stabilization, and ERK inactivation upon HDI treatment reduces the E1A protein level. Our results indicate that the combination treatment of tumors with activated Ras using adenoviral E1A and HDI has limitations attributed to intense HDI-dependent degradation of E1A. Nevertheless, the established contribution of ERK kinase to the regulation of E1A stability can be used to search for new effective drug combinations based on the adenoviral E1A protein.

## INTRODUCTION

The early region 1A (*E1A*) gene of human adenovirus type 5 (Ad5) is the first gene to be expressed during adenovirus infection, and the E1A protein is a critical regulator of viral replication. The E1A protein orchestrates the expression of other adenoviral genes and ensures the necessary conditions for viral replication; namely, it stimulates the transition of infected cells to the S phase of the cell cycle (DNA synthesis phase) [1]. E1A *per se *cannot directly interact with DNA, but, being a cofactor of many transcription factors and coactivators, it alters the activity of such proteins as Rb, the inhibitor of cyclin-dependent kinases p21/Waf; acetyltransferase CBP/p300; the transcription factors ATF, AP1, Sp1, etc. [2]. Despite the viral nature of E1A, its scientific significance goes far beyond virology. Expression of the *E1A *gene immortalizes primary cells due to the stimulation of S-phase progression and suppression of replicative senescence [3]. In primary rodent cells, E1A promotes oncogenic transformation in combination with activated *ras *[4] or other complementary oncogenes (e.g., another Ad5 early region gene *E1B*) [5]. However, E1A is not oncogenic in human cells [6, 7].

Abundant experimental data points to the tumor- suppressive properties of E1A in various types of human cancer cells: carcinoma, fibrosarcoma, and melanoma. These observations seem surprising, given the diversity of genetic changes in these three types of tumors. Several mechanisms of E1A-mediated tumor growth suppression have been established, including the reduction of the metastatic potential, as well as apoptosis induction [8, 9].

Later studies have shown that *E1A *expression increases the sensitivity of cancer cells to a number of cytotoxic agents used in antitumor therapy, such as etoposide, cisplatin, taxanes, etc. [10, 11]. It should be noted that adenoviral E1A selectively sensitizes multiple cancer cells, but not normal cells [12, 13]. Therefore, adenoviral E1A is considered a promising sensitizing component of combination cancer therapy.

We investigated the possibility of using E1A in combination chemotherapy with histone deacetylase inhibitors (HDIs). HDI aims at increasing histone acetylation, which is an epigenetic modification regulating such fundamental cellular processes as gene expression, DNA replication, and genome stability [14]. It has been shown that E1A sensitizes tumor cells with respect to HDIs (SAHA, TSA) more effectively than with respect to other chemotherapy drugs (5-fluorouracil, cisplatin, etoposide, or paclitaxel) [13]. However, as we have shown earlier, HDI induced degradation of E1A [15].

In our study, we used sodium butyrate, which is a broad-spectrum HDI inhibiting all histone deacetylases, except for HDAC6 and HDAC10 belonging to class II and class III histone deacetylases, respectively [16]. Sodium butyrate is a natural metabolite formed in the mammalian body [17]. Therefore, it has low cytotoxicity against normal cells and selectively kills cancer cells [18].

The E1A protein, like the products of other oncogenes, has a short half-life of approximately 40 min [19]. Normally, the intracellular level of regulatory proteins with a short half-life, such as cyclins, p53, beta- catenin, p27/kip and Myc, is controlled by the ubiquitin-proteasome system. Accordingly, it can be assumed that the E1A protein is degraded by the same mechanism. However, the exact pathways for the E1A stability regulation have not yet been elucidated. It has been shown that degradation of the E1A protein is triggered through phosphorylation of its C-terminal amino acid residues rather than through ubiquitination [20]. Notably, the E1A protein itself acts as a proteasome regulator that can both suppress the ubiquitin– proteasome system by direct binding of its N-terminal region to the 26S proteasome subunit [20] and to stimulate the ubiquitination of individual proteins [21].

Previously, we showed that there was a difference in the dynamics of HDI-induced E1A degradation in cells expressing wild-type Ras or a mutant Ras protein [15]. These observations suggest that there is a role played by the Ras protein in the regulation of E1A stability. The small GTPase Ras is a key regulator of cell growth [22]. Normally, Ras is activated in response to extracellular stimuli and initiates the proliferation programs. However, some pathologies are accompanied by constitutive activation of the Ras protein, leading to the permanent activation of underlying Ras-dependent signaling pathways, which results in cell division independent of environmental signals and carcinogenesis induction [23]. Ras gene mutations leading to a constitutive activity of the Ras protein have been found in many tumor types, including aggressive and difficult-to-treat cancers such as melanoma, colorectal cancer, and lung cancer [24]. Therefore, searching for therapy methods for tumors carrying Ras mutations is critical in molecular biology.

The aim of this study is to reveal the role of activated Ras in the regulation of E1A stability in untreated or HDI-treated cells in order to determine the rationality of combination therapy with E1A and HDI for treating Ras-mutated tumors.

## MATERIALS AND METHODS

**Cell lines**

The E1A+Ras cell line was obtained by transformation of mouse embryonic fibroblasts with complementary oncogenes: the early region E1A gene of human adenovirus type 5 (Ad5) and cHa-ras carrying the activating mutations in codons 12 and 61 [25]. The E1A+E1B line was obtained by transformation of rat embryonic fibroblasts with the Ad5 HindIII region encoding the E1A and E1B proteins. Human embryonic kidney cells transformed with adenovirus type 5 (HEK293) were obtained from the Center for Collective Use “Collection of Vertebrate Cell Cultures”.

The cells were cultured at 37°C and 5% CO_2_ in a DMEM medium supplemented with 10% FCS. The cells were treated with 4 mM sodium butyrate (Calbiochem, USA) and/or 1–2 μM lactacystin (Calbiochem).

## RT-PCR

RNA was isolated from the cells using the Trizol reagent (Invitrogen, USA). Reverse transcription was performed with 2 µg of RNA and 1 µg of random hexaprimers. The PCR reaction was carried out on a PCR cycler (Eppendorf Mastercycler Personal, AG 22331) in the presence of 100 ng primers to cDNA of the genes of interest (*E1A*: 5´-CTTTCCACCCAGTGACGACG- 3 ´ / 5 ´ -TGTCGGGCGTCTCAGGATAG - 3 ´ ;* gapdh*: 5´-TCATCAGCAATGCCTCCTGCACC-3´/5´- ACAGTTTCCCGGAGGGGCCA-3´) for 22–32 cycles: denaturation for 30 s (95°C), primer annealing for 30 s (61°C E1A, 58°C gapdh), and elongation for 1 min (72°C).

**Fractionation of cell extracts**

The cells were suspended in 10 mM HEPES-KOH (pH 7.9); 0.4% NP-40 was then added. The cells were centrifuged at 5,000 rpm to obtain cytoplasmic extracts. The pellets were lysed in 20 mM HEPES-KOH (pH 7.9) and then centrifuged at 15,000 rpm to obtain nuclear extracts.

**Immunoprecipitation and immunoblotting**

The cells were lysed in a buffer containing 0.5% NP-40, 1% Triton X-100, protease and phosphatase inhibitors (a buffer containing 1% NP-40, 0.5% sodium deoxycholate, 0.1% sodium dodecyl sulfate (SDS) was used for immunoprecipitation). Proteins were separated in a 10–12% polyacrylamide gel, transferred to a PVDF membrane (Millipore, USA), and analyzed with specific antibodies, detected by enhanced chemiluminescence (ECL, Amersham Biosciences, UK) and visualized using a Syngene PXi6 Access system. We used antibodies against proteins E1A sc-25 G1713 1 : 1000 (Santa Cruz Biotechnology, Inc., USA), pan-Ras OP40 1 : 1000 (Calbiochem), pERK1/2 #4377 1 : 800 (Cell Signaling, USA), pAkt (Ser 473) #4060 1 : 1000 (Cell Signaling), p-p38 #9211 1 : 1000 (Cell Signaling), p-JNK #9251 1 : 500 (Cell Signaling), acetylated lysine #9441 1 : 500 (Cell Signaling ), α-tubulin sc-32293 1 : 10000 (Santa Cruz Biotechnology, Inc.), and Gapdh 2118 1 : 1000 (Cell Signaling). Immunoblotting for each protein was performed at least in triplicate. The ImageJ software was used for densitometric analysis. The diagrams show the values normalized to the loading control (Gapdh) and reduced to relative units of measurement. The diagrams show the average values for the 3–5 experiments; the error bars represent the standard error of the mean (SEM).

**Transient transfection**

For transfection, the cells were plated onto a 12-well plate (DMEM supplemented with 10% FCS without antibiotic) at a density of 150 × 10^3^ cells per well. Transfection of pcDNA3 (Addgene) and pSV2-ras vectors encoding cHa-ras (Addgene) was performed with Lipofectamine-2000 (Invitrogen) according to the manufacturer’s protocol.

## RESULTS

**The influence of HDI sodium butyrate on the dynamics of E1A degradation in cells with different Ras protein status**

To study the impact of the Ras signaling pathway on the E1A stability, we used two E1A-expressing transformed cell lines differing in the activity status of the Ras protein: the E1A+Ras cell line expressing cHa-*ras* with activating mutation and the E1A+E1B line expressing wild-type *ras*.

**Fig. 1:**
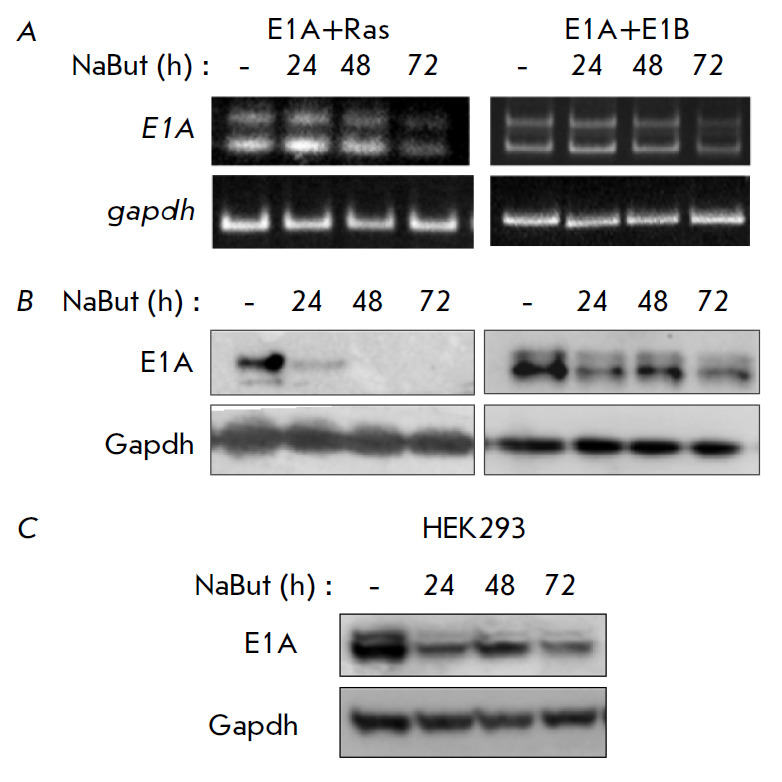
NaBut causes the degradation of the E1A protein, which is most pronounced in cells with activated Ras. (*A*) Analysis of e1a transcription by RT-PCR. Amount of the E1A protein product (immunoblotting) in E1A-expressing rodent (*B*) and human (*C*) cells. The gapdh gene and its protein product were used as a loading control in RT-PCR and immunoblotting, respectively

The RT-PCR and immunoblotting data show that sodium butyrate (NaBut) does not affect the transcription of the *E1A *gene (*Fig. 1A*), while its protein product is degraded in both cell lines, but with different dynamics and intensities (*Fig. 1B*). In E1A+Ras cells, the E1A protein degrades rapidly under NaBut treatment. Whereas E1A can be detected even after 72 h of exposure of E1A+E1B cells to NaBut. Similar dynamics of the moderate decline in the E1A expression upon treatment with NaBut is also observed in HEK293 cells expressing the wild-type *ras *gene (*Fig. 1C*).

**Expression of activated Ras increases the E1A protein level but leads to E1A destabilization upon treatment with sodium butyrate**

**Fig. 2:**
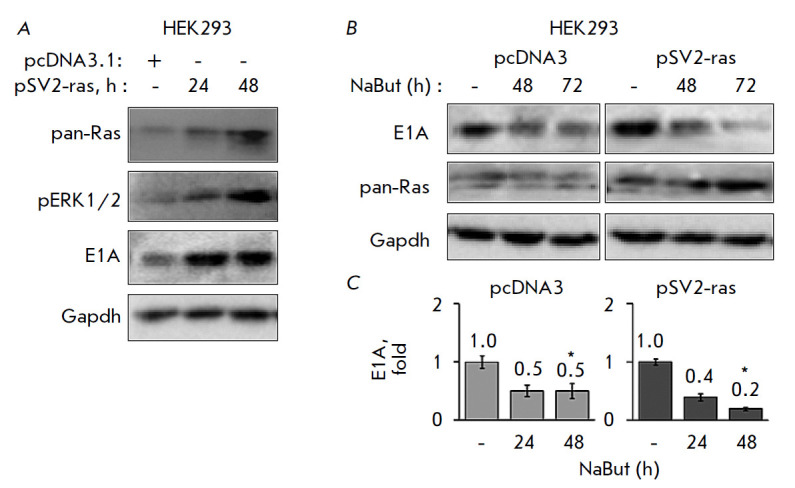
Activated Ras stabilizes E1A, but also enhances its degradation under the action of NaBut. Immunoblotting of proteins from HEK293 cells (*A*) transfected with pcDNA3 (control vector) or pSV2-ras vectors with antibodies against E1A, pERK, and pan-Ras and (*B*) transfected with pcDNA3 (control vector) or pSV2-ras vectors, and treated with 4 mM NaBut for 0–72 h, with antibodies against E1A and pan-Ras. Gapdh is used as the loading control. (*C*) Bar plots of the average E1A level in transfected HEK293 cells under the action of NaBut, obtained by densitometric analysis of loading- control-normalized (Gapdh) immunoblotting data; the amount of E1A in untreated cells is taken to be unity. Error bars are based on the standard error of the mean (SEM). The Mann–Whitney test was used for comparing the values for two vectors within each timepoint (*p < 0.05)

To confirm the role played by activated Ras in the regulation of the E1A protein stability, an expression vector encoding cHa-Ras with activating mutations was introduced into HEK293 cells. Immunoblotting reveals an increased phosphorylation state of MAP kinase ERK in cells transfected with mutant cHa-ras, compared to that in cells transfected with a control vector pcDNA3, thus confirming the activated state of exogenous Ras (*Fig. 2A*). Expression of activated Ras is accompanied by the accumulation of the adenoviral E1A protein (*Fig. 2A*). Thus, our results show a stabilizing effect of activated Ras signaling on the adenovirus E1A protein.

According to the immunoblotting data, the adenoviral E1A protein degrades faster upon exposure to NaBut in cells transfected with mutant cHa-ras than in cells transfected with the control vector pcDNA3 (*Fig. 2 B,C*). Thus, overexpression of activated Ras leads to the accumulation of the E1A protein but makes E1A more sensitive to NaBut-induced degradation.

**Fig. 3:**
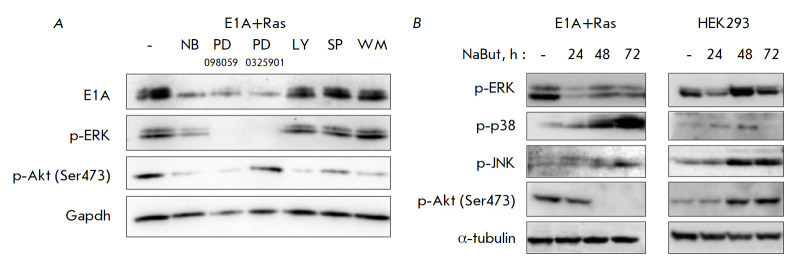
Akt and ERK kinases as E1A stabilizing factors in Ras-expressing cells. (*A*) The dynamics of the E1A protein product in Ras-activated E1A-expressing cells upon treatment with 4 mM NaBut and inhibitors of Ras-dependent kinases (50 μM PD098059 and PD0325901 – ERK inhibitors, 20 μM LY and 10 μM WM – PI3K inhibitors, 10 μM SP – JNK inhibitor) for 24 h. (*B*) The dynamics of kinase phosphorylation under the action of NaBut in cells with activated and normal Ras. Immunoblotting of proteins from E1A+Ras and HEK293 cells untreated or treated with 4 mM NaBut for 0–48 h. Gapdh/α-tubulin are used as a loading control

The mechanisms of Ras-dependent E1A stabilization were identified using chemical inhibitors of the downstream kinases in the Ras signaling pathways. Immunoblotting reveals that suppression of exclusively ERK kinase activity by specific inhibitors PD098059 or PD0325901 leads to the destabilization of E1A in E1A+Ras cells, like in the case of NaBut (*Fig. 3A*).

To elucidate the mechanisms of HDI-induced E1A protein degradation, we compared the effect of NaBut on the activity/phosphorylation status of various Ras-dependent kinases in cells with different Ras status. According to immunoblotting with phosphospecific antibodies, NaBut changes the activity of the p38 and JNK kinases in cells with normal and activated Ras in a similar manner, whereas NaBut affects the activities of the ERK and PKB/Akt kinases differently, depending on the Ras status in the cell (*Fig. 3B*). Therefore, NaBut reduces the activity of the ERK and PKB/Akt kinases in E1A+Ras cells with activated Ras, while activity of these kinases increases in HEK293 cells expressing normal Ras (*Fig. 3B*). These data imply the involvement of the ERK and PKB/Akt kinases in the regulation of both the basal E1A protein level and NaBut-induced decline of the E1A protein level.

**Proteasome inhibition does not abolish NaBut-induced E1A level reduction**

**Fig. 4:**
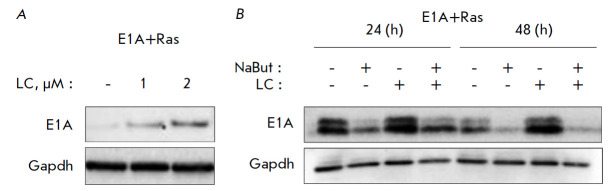
Immunoblotting of E1A+Ras cells (*A*) treated with the proteasome inhibitor lactacystin (1 μM and 2 μM LC) or (*B*) co-treated with NaBut and/or 2 μM LC, with anti-E1A antibodies, for 24–48 h. Gapdh is used as a loading control

In order to reveal the role of the ubiquitin–proteasome system in the HDI-dependent reduction of the E1A level, E1A+Ras cells were treated with a proteasome inhibitor lactacystin (LC). LC treatment was accompanied by a dose-dependent increase in E1A protein level (*Fig. 4A*).

To test the possibility of preventing the NaButinduced degradation of E1A by suppressing proteasome activity, E1A+Ras cells were treated with either NaBut or its combination with LC for 24–48 h. Immunoblotting data showed that after 24 h, LC had a slight stabilizing effect on both the control and NaBut-treated cells; however, upon prolonged exposure the amount of the E1A protein decreased regardless of the presence of LC (*Fig. 4B*).

Therefore, we have shown that LC increases the basal level of the E1A protein but does not prevent its degradation during a prolonged action of NaBut.

**Fig. 5:**
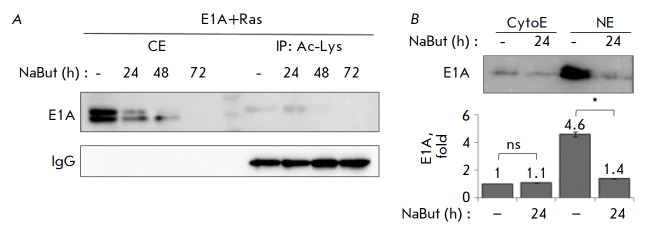
Acetylation and relocalization of E1A under the action of NaBut. (*A*) Immunoprecipitation with acetylated lysine antibodies (IP: Ac-Lys), followed by immunoblotting with anti-E1A antibodies. Immunoglobulins G (IgG) were used as a loading control. (*B*) Immunoblotting of fractionated cell extracts (CytoE – cytoplasmic extracts, NE – nuclear extracts) with anti-E1A antibodies. The extracts were obtained from cells either untreated or treated with 4 mM NaBut for 24 h. The bar plot shows the average amount of E1A obtained by densitometric analysis of the immunoblotting data normalized to lane loading signal intensity (Ponceau S); the amount of E1A in the untreated NaBut cytoplasmic extract was taken to be unity. Error bars are based on the standard error of the mean (SEM). The Mann–Whitney test was used to check the significance of the differences (ns *p *> 0.05, *p < 0.05)

For protein degradation to occur, the protein needs to be located in the cytoplasm. It was shown previously that E1A relocalization can be affected by its acetylation [26]. In this regard, the effect of NaBut on the E1A acetylation level and its intracellular localization was studied. According to the results of immunoblotting performed after immunoprecipitation with acetylated-lysine antibodies, NaBut causes the accumulation of acetylated E1A in E1A+Ras cells during the first 24 h, but then the E1A protein is no longer detected (*Fig. 5A*). Meanwhile, the immunoblotting data for fractionated cell extracts indicate that the E1A protein, which is predominantly localized in the nucleus, is released from it under the action of NaBut (*Fig. 5B*). This suggests that NaBut enhances the acetylation of the E1A protein, thus leading to its relocalization from the nucleus to the cytoplasm, where it undergoes rapid degradation.

## DISCUSSION

The ability of HDI to cause degradation of the adenoviral E1A protein has been demonstrated [13, 26, 27, 28], but the mechanisms of E1A degradation, as well as E1A stabilization factors, have not yet been elucidated. We have previously shown that HDIs sodium butyrate, trichostatin A, and vorinostat (SAHA) cause degradation of the adenoviral E1A protein, while the dynamics of reduction of the HDI-induced E1A level correlates with the activity of the Ras protein in cells [29].

In this paper, we studied the contribution of Rassignaling pathway proteins to the stability of adenoviral E1A. We have demonstrated for the first time that overexpression of activated Ras leads to an accumulation of the E1A protein. According to our data, ERK1/2 kinases play a decisive role in the Rasdependent stabilization of E1A. Thus, the accumula tion of the adenoviral E1A protein induced by overexpression of activated Ras is accompanied by ERK1/2 activation (*Fig. 2*) and the suppression of the MEK/ ERK pathway activity by pharmacological inhibitors reduces the E1A level (*Fig. 3B*).

HDI-induced degradation of E1A is also mediated by ERK kinases. The HDI-induced decrease in the E1A protein level is accompanied by inactivation of ERK kinase (*Fig. 3A*). NaBut also inactivates PKB/ Akt kinase in cells with activated Ras. However, reduction of the PKB/Akt activity does not affect E1A expression, as demonstrated in the experiments using specific Akt inhibitors (*Fig. 3A*).

The involvement of Ras signaling in the E1A regulation is not surprising, since during infection, viruses induce signal transduction through the MAP kinase cascade [30] and, in particular, through the ERK kinase [31]. It is known that adenovirus enhances ERK activity both in the early and late phases of the infection [32].

Understanding the interplay between the virus and the Ras signaling pathway can be crucial for constructing oncolytic viruses replicating specifically in cancer cells, as well as for developing new adenovirus- based strategies for cancer therapy.

Phosphorylation at serine residues plays an important role in the regulation of E1A protein activity. Thus, ERK1/2-mediated phosphorylation of E1A at the Ser185 and Ser188 residues increases gene expression from the E4 promoter [33]. However, the role of phosphorylation in the stability of the E1A protein has not been sufficiently studied yet. So far, only two studies have shown that both the expression and functions of the E1A protein are strongly dependent on the MEK/ERK kinase cascade [32, 33]. Meanwhile, it is assumed that the Ras/MEK/ ERK signaling pathway affects the efficiency of E1A translation rather than the rate of E1A protein degradation.

Using the proteasome inhibitor lactacystin, we found that the basal level of E1A protein increases under exposure to a proteasome inhibitor, thus confirming that E1A is normally utilized through the proteasome pathway; these findings are consistent with the results demonstrating the role played by proteasomes in the degradation of E1A isoforms [34]. However, lactacystin did not abolish the HDIinduced reduction in the E1A protein level, in contrast to the basal level of the E1A protein, thus suggesting that HDI-dependent degradation of E1A occurs not through the ubiquitin-proteasome pathway, but rather through an alternative mechanism of E1A destabilization induced by HDI in Ras-transformed cells. Sodium butyrate, an inhibitor of a wide class of histone deacetylase, can also use non-histone proteins as a substrate, and, accordingly, affect the level of E1A protein acetylation. The E1A protein is acetylated at Lys239 in the C-terminal domain by acetyltransferases CBP, p300, and pCAF, which impedes the nuclear localization of E1A through impaired binding to importin- α [26], making E1A accessible to degradation systems.

It is known that constant activation of the Ras signaling pathway leads to the induction of the transcription factor HSF1, which controls the expression of heat shock proteins [35], which allows one to suggest that the Hsp-dependent degradation mechanism [36] might be involved in the HDI-induced destabilization of the E1A protein. However, the contribution of chaperone-mediated autophagy to the utilization of the E1A protein requires further research.

## CONCLUSIONS

1. Activated Ras stabilizes E1A through the activation of downstream kinase ERK.

2. The E1A protein level drops significantly after exposure to NaBut in cells with activated Ras as a result of HDI-dependent inactivation of ERK kinase.

3. Normally, E1A is utilized in proteasome degradation; however, under a prolonged action of sodium butyrate, E1A degradation is observed even upon proteasome inhibition, which means that HDI-dependent degradation of E1A does not occur via the ubiquitin- proteasome pathway.

4. HDI-induced degradation of E1A, which was shown to take place in cells with activated Ras, implies that the application of combination therapy with E1A and HDI in the treatment of tumors with mutant Ras is limited.

## Abbreviations

HDI: histone deacetylase inhibitors
E1A: protein encoded by the adenovirus early region 1A gene
LC: lactacystin (a proteasome inhibitor)
WM: wortmannin (a PI3 kinase inhibitor)
NaBut: sodium butyrate
Ac-Lys: acetylated lysine
